# Molecular surveillance of dengue virus in field-collected *Aedes* mosquitoes from Bhopal, central India: evidence of circulation of a new lineage of serotype 2

**DOI:** 10.3389/fmicb.2023.1260812

**Published:** 2023-09-14

**Authors:** Devojit Kumar Sarma, Lokendra Rathod, Sweta Mishra, Deepanker Das, Ankita Agarwal, Gaurav Sharma, Tanim Arpit Singh, Manoj Kumawat, Samradhi Singh, Vinod Verma, Manoj Kumar, Swasti Shubham, Rajnarayan R. Tiwari, Anil Prakash

**Affiliations:** ^1^ICMR-National Institute for Research in Environmental Health, Bhopal, India; ^2^State Virology Laboratory, Department of Microbiology, Gandhi Medical College, Bhopal, India; ^3^Maharaja Ranjit Singh College of Professional Sciences, Indore, India; ^4^Sanjay Gandhi Postgraduate Institute of Medical Sciences, Lucknow, India

**Keywords:** dengue virus, *Aedes* mosquito, surveillance, epidemiology, India font: Italic, complex script font: Italic considering the growing threat

## Abstract

**Introduction:**

Dengue fever is hyperendemic in several Southeast and South Asian countries, including India, with all four serotypes (DENV 1–4) circulating at different periods and in different locations. Sustainable and improved virological and entomological surveillance is the only tool to prevent dengue and other vector-borne diseases.

**Objectives:**

The present study has been carried out to detect and characterize the circulating dengue virus (DENV) in field-collected *Aedes* mosquitoes in Bhopal, Central India.

**Methods:**

*Aedes* mosquitoes were collected from 29 localities within Bhopal city during October 2020 to September 2022. DENV infection was assessed in the individual head and thorax regions of *Aedes* mosquitoes using reverse transcriptase PCR. Positive samples were sequenced, and the circulating serotypes and genotypes were determined using phylogenetic analysis.

**Results:**

DENV RNA was detected in 7 *Aedes aegypti* and 1 *Aedes albopictus*, with infection rates of 0.59 and 0.14%, respectively. Phylogenetic analysis revealed all the isolates belonged to DENV serotype 2 and distinctly clustered with the non-Indian lineage (cosmopolitan genotype 4a), which was not recorded from the study area earlier. The time to most common recent ancestor (TMRCA) of these sequences was 7.4 years old, with the highest posterior density (HPD) of 3.5–12.2 years, indicating that this new lineage emerged during the year 2014. This is the first report on the DENV incrimination in both *Ae. aegypti* and *Ae. albopictus* mosquitoes collected from Bhopal, Central India.

**Conclusion:**

The observed emergence of the non-Indian lineage of DENV-2 in Bhopal, which again is a first report from the area, coincides with the gradual increase in DENV cases in Bhopal since 2014. This study emphasizes the importance of DENV surveillance and risk assessment in this strategically important part of the country to decipher its outbreak and severe disease-causing potential.

## 1. Introduction

Dengue fever is caused by the dengue virus (DENV), a single-stranded positive-sense RNA virus of the *Flaviviridae* family. It is the most recognized arbovirus in the world, transmitted by infected female mosquitoes, especially *Aedes aegypti* and *Ae. albopictus* (Hayes and Gubler, 1992). Dengue infections have increased exponentially in the majority of the tropics and subtropics over the past three decades. Currently, one third of the world’s population is susceptible to DENV infection. The WHO estimates that the global burden of dengue infection has increased about 10.3-fold in the last two decades (World Health Organization, 2023). This condition is primarily linked to uncontrolled urbanization, climate change, poor water supply and sewage management, rapid movement of people, animals, and trade via air and sea routes, and unsustainable vector control programmes (Wilder-Smith and Gubler, 2008; Gubler, 2011; Bhatia et al., 2022; Romanello et al., 2022).

Dengue infection is caused by four antigenically different serotypes: DENV-1, DENV-2, DENV-3, and DENV-4 (Gubler, 2002) and the disease manifests itself in a range of ways, from asymptomatic infection and mild febrile illness (dengue fever) to more severe forms including dengue hemorrhagic fever (DHF) and dengue shock syndrome (DSS). The most severe clinical manifestation, dengue shock syndrome (DSS), is characterized by coagulation abnormalities, hemorrhage, plasma leakage, and organ failure (Bhatt et al., 2021). The present dengue case classification, according to World Health Organization, comprises symptomatic patients with and without warning signals, as well as severe dengue (World Health Organization, 2009). Based on the genetic make-up of DENV each serotype has been subdivided into 4–5 genotypes (Weaver and Vasilakis, 2009). Furthermore, depending on their phylogenetic classification, each genotype has a range of lineages (Shrivastava et al., 2015). Infection with one serotype results in lifetime immunity against homologous serotypes, while there is no or limited immunity against infection to heterologous dengue serotypes. This secondary infection, which leads to enhanced dengue severity, is primarily caused by antibody-dependent enhancement (ADE), a mechanism mediated mostly by immunoglobulin G (IgG) (Teo et al., 2023). Additionally, certain lineages and genotypes have been associated with severe forms of dengue fever. These genetic variants with modest variations are also responsible for major outbreaks due to rapid transmission in both humans and mosquitoes (Messer et al., 2003). Hence, regular surveillance is necessary for the control and management of dengue.

Recent anthropogenic and climate-related changes have resulted in accelerated transmission of DENV, causing frequent dengue epidemics (Sarma et al., 2022). Previous studies suggested that several Southeast Asian countries demonstrated dengue hyperendemicity that was 18-fold higher than the Americas (Murray et al., 2013). Currently, almost all Indian states are under constant threat of dengue transmission. A nationwide dengue sero-survey during 2017–2018 showed that nearly half of the Indian population (48.7; 95% CI 43.5–54.0) was sero-positive for DENV (Murhekar et al., 2019). Since 2000, the north Indian states, including New Delhi, Rajasthan, Uttar Pradesh, Madhya Pradesh, Haryana, and Punjab, have reported dengue outbreaks at regular intervals (Mishra et al., 2015). Similarly, the western (Maharashtra), eastern (Odisha), and southern states (Kerala, Andhra Pradesh, and Telangana) also reported massive dengue outbreaks along with chikungunya (Anoop et al., 2010; Cecilia et al., 2011; Shrivastava et al., 2015). In view of this, there is an increasing demand for a safe and effective DENV vaccine that elicits immunity against all the four serotypes. At the moment, at least seven DENV vaccines are at various stages of clinical trials or pre-clinical investigations. Three of these, Dengvaxia^®^ (CYT-TDV), Qdenga^®^ (TAK-003) and TV003 (NIAD/Butantan/Merck) have shown promising results in clinical trials (Torres-Flores et al., 2022; Angelin et al., 2023). Dengvaxia^®^, manufactured by Sanofi Pasteur, has been licensed in 20 countries and approved for use in individuals aged 6–45 years with laboratory confirmed previous dengue infection and living in endemic countries (Thomas and Yoon, 2019; Torres-Flores et al., 2022). However, shortcomings such as vaccine administration limited to dengue immune individuals only, non-availability in non-endemic countries and safety concerns in vaccine recipients who were dengue non-immune at the time of vaccine administration (Hadinegoro et al., 2015) in the case of Dengvaxia^®^ and less protection against DENV-3 (Rivera et al., 2022), as well as a lack of data for elderly individuals (Angelin et al., 2023) in the case of Qdenga^®^, have posed significant challenges to the widespread and effective control of dengue fever through vaccination efforts. Until an effective vaccine becomes available, vector control will continue to be the primary approach for managing dengue transmission. Furthermore, systematic molecular monitoring of circulating DENV serotypes in mosquitoes and humans, as well as early detection of any serotype or genotype shift in a geographic area, would aid in the prediction of imminent DENV epidemics and devise targeted public health strategies for dengue control.

This study investigated the phylogenetic relationship of DENV-2 serotypes prevalent in central India (Bhopal, Madhya Pradesh) and reported the emergence of a new lineage (Genotype 4a, cosmopolitan non-Indian lineage) in naturally infected, wild-caught *Ae. aegypti* and *Ae. albopictus* mosquitoes. This study highlights the urgent need of DENV surveillance and clinical characterization of dengue fever in this strategically located part of the country.

## 2. Materials and methods

### 2.1. Collection, identification, and pooling of *Aedes* mosquito larvae

**FIGURE 1:**
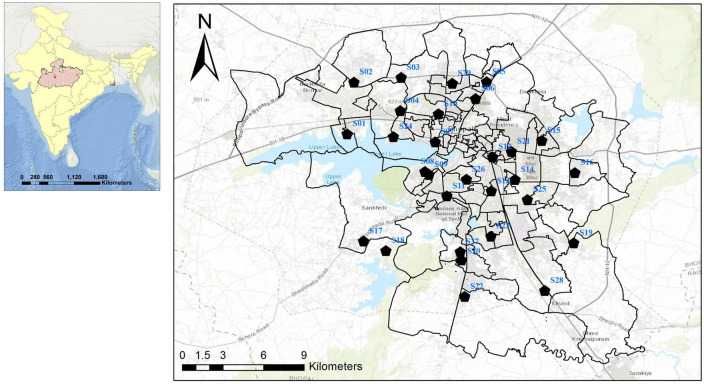
Map of Bhopal city showing *Aedes* spp. mosquito collection points.

The study was conducted in Bhopal, the capital city of Madhya Pradesh in central India. House-frequenting adult *Aedes* mosquitoes were collected from October 2020 to September 2022 during morning and evening hours from 29 different locations in Bhopal (Figure 1). Mosquitoes were collected using mechanical aspirators and transported to the laboratory, and then identified to species level using standard taxonomic keys (Tyagi et al., 2015). Each female individual of *Ae. aegypti* and *Ae. albopictus* was bisected into the head, thorax, and abdomen and stored separately in 1.5 mL micro-centrifuge tubes filled with 50 μl of RNAlater™ stabilization solution (Sigma-Aldrich, Cat. # R0901-100ML) at −20°C till further processing. As some of the adult mosquitoes were damaged during transportation, their head and thorax could not be bisected and hence were not processed further. *Aedes* immatures were also collected from the 29 localities in different larval habitats. The collected larvae were transferred to the lab and reared up to the 4th instar. A total of 10 larvae were kept separately in 1.5-ml micro-centrifuge tubes filled with 100 μl of RNAlater™ stabilization solution and stored at −20°C till further processing. The remaining larvae were reared up to adult stage, identified, and stored at 4°C.

### 2.2. RNA extraction, DENV diagnosis, and sequencing

RNA was extracted from individual mosquito’s head-thorax region and from larvae pool (10 larvae/pool) using the Qiagen Viral RNA Mini Kit (Qiagen, cat. # 52906), according to the manufacturer’s instructions. DENV infection in the extracted RNA of individual mosquitoes, as well as larval pool was assessed by one-step reverse transcription-PCR (Thermo Fisher Scientific, Cat. # 12594025) targeting the capsid-pre-membrane (*C-prM*) gene region with primers and methods as described (Lanciotti et al., 1992). The 511-bp PCR product of the DENV-positive individuals was gel purified, sequenced in both directions, and used to identify and characterize circulating serotypes and genotypes.

### 2.3. Phylogenetic analysis

The DENV sequences generated in this study were aligned with representative DENV *C-prM* sequences from India and other countries (Supplementary Table 1) using the MUSCLE programme, and a maximum likelihood phylogenetic tree with 1000 bootstrap replicates was reconstructed using the MEGA10 software (Kumar et al., 2018) to identify the serotypes and genotypes of the DENV isolates. These sequences have been submitted to GenBank with the accession numbers OQ842497-OQ842504. The genetic distance within and between the genotypes was calculated using MEGA10 software. A maximum clade credibility (MCC) tree was used to determine the time to the most recent common ancestor (TMRCA) using DENV-2 genotype 4a and 4b sequences, with sylvatic sequences as the outgroup, using the BEAST 2.5 package (Bouckaert et al., 2019). Based on the lowest BIC (Bayesian Information Criterion) scores, Kimura 2 parameter with discrete gamma distribution (K2 + G) model, assessed through MEGA10 software, was used as the best fit model for Bayesian Markov Chain Monte Carlo (MCMC) analysis. Relaxed uncorrelated lognormal molecular clock model was used and an effective population size of >200 was ensured by running MCMC chains for 4E08 generations with a sampling frequency of 10000 and burn-in of 1000. Tracer v1.7.2, TreeAnnotator v1.10.4 and FigTree v1.4.4 was used to analyze the output and view and annotate the MCC tree.

### 2.4. Ethical approval

The present study was approved by the Institutional Ethics Committee of the ICMR-National Institute for Research in Environmental Health, Bhopal (NIREH/BPL/IEC/2018-19/3130, dated March 18, 2019).

## 3. Results

A total of 2,371 adult female *Aedes* mosquitoes (1,498 *Ae. aegypti* and 873 *Ae. albopictus*) were collected. Of these, 1,890 mosquitoes (1,186 *Ae. aegypti* and 704 *Ae. albopictus*) were tested for DENV infection. Similarly, 370 *Aedes* larvae were tested for DENV infection in 37 pools (32 pools for *Ae. aegypti* and 5 pools for *Ae. albopictus*). Based on the RT-PCR amplification of *C-PrM* gene region (Supplementary Figure 1), DENV RNA was detected in seven *Ae. aegypti* and one *Ae. albopictus* individuals. Overall DENV infection rates were 0.59 and 0.14% in *Ae. aegypti* and *Ae. albopictus*, respectively. Area wise S_15 (Nizamuddin colony area) recorded the highest DENV infection rate (5.26%), while S_11 (Kotra Sultanabad area) recorded the lowest DENV infection rate (1.25%) for *Ae. aegypti* mosquitoes. *Ae. albopictus* was incriminated only in S_25 (Shakti Nagar) with an infection rate of 0.8% (Table 1). No DENV infection was detected in larval pools.

**TABLE 1:** *Aedes* mosquitoes collected, processed and tested positive for DENV in Bhopal city.

Site no.	Site name	*Aedes aegypti*				*Aedes albopictus*			
		Number collected	Number tested for DENV infection	Number found positive for DENV infection	Infection rate (%)	Number collected	Number tested for DENV infection	Number. found positive for DENV infection	Infection rate (%)
S_01	Bairagarh	54	46	0	0.0	2	0	0	0
S_02	Gandhi Nagar	93	87	0	0.0	11	4	0	0
S_03	Sanjiv Nagar	40	19	0	0.0	82	75	0	0
S_04	Lalghati	88	70	1	1.42	13	0	0	0
S_05	Bhanpur	26	14	0	0.0	3	0	0	0
S_06	Gupta Nagar	30	13	0	0.0	15	9	0	0
S_07	Shaid Nagar	43	30	0	0.0	2	0	0	0
S_08	NITTR	28	21	0	0.0	99	88	0	0
S_09	Banganga	38	20	0	0.0	25	24	0	0
S_10	Jamalpura	66	55	1	1.81	4	0	0	0
S_11	Kotra Sultanabad	86	80	1	1.25	157	141	0	0
S_12	Subhash Nagar	81	67	0	0.0	1	0	0	0
S_13	Shankar Nagar	192	181	0	0.0	14	10	0	0
S_14	Anna Nagar	50	38	0	0.0	7	4	0	0
S_15	Nizamuddin Colony	45	38	2	5.26	6	0	0	0
S_16	Awadhpuri	49	41	0	0.0	7	4	0	0
S_17	Neelbad	3	0	0	0.0	31	29	0	0
S_18	NLU	19	12	0	0.0	30	19	0	0
S_19	Katara Hills	18	5	0	0.0	86	76	0	0
S_20	Mahabali Nagar	48	38	0	0.0	7	5	0	0
S_21	BHEL Area	19	15	0	0.0	35	25	0	0
S_22	Priyanka Nagar	33	31	0	0.0	1	0	0	0
S_23	Gulmohar	34	22	0	0.0	11	6	0	0
S_24	Khanugaon	26	16	0	0.0	19	13	0	0
S_25	Shakti Nagar	28	16	0	0.0	139	124	1	0.8
S_26	Bheem Nagar	36	25	0	0.0	12	7	0	0
S_27	Kolar Road	97	86	2	2.32	2	0	0	0
S_28	Misroad	27	14	0	0.0	44	36	0	0
S_29	Karond	101	86	0	0.0	8	5	0	0
Total		1498	1186	7	0.59	873	704	1	0.14

**FIGURE 2:**
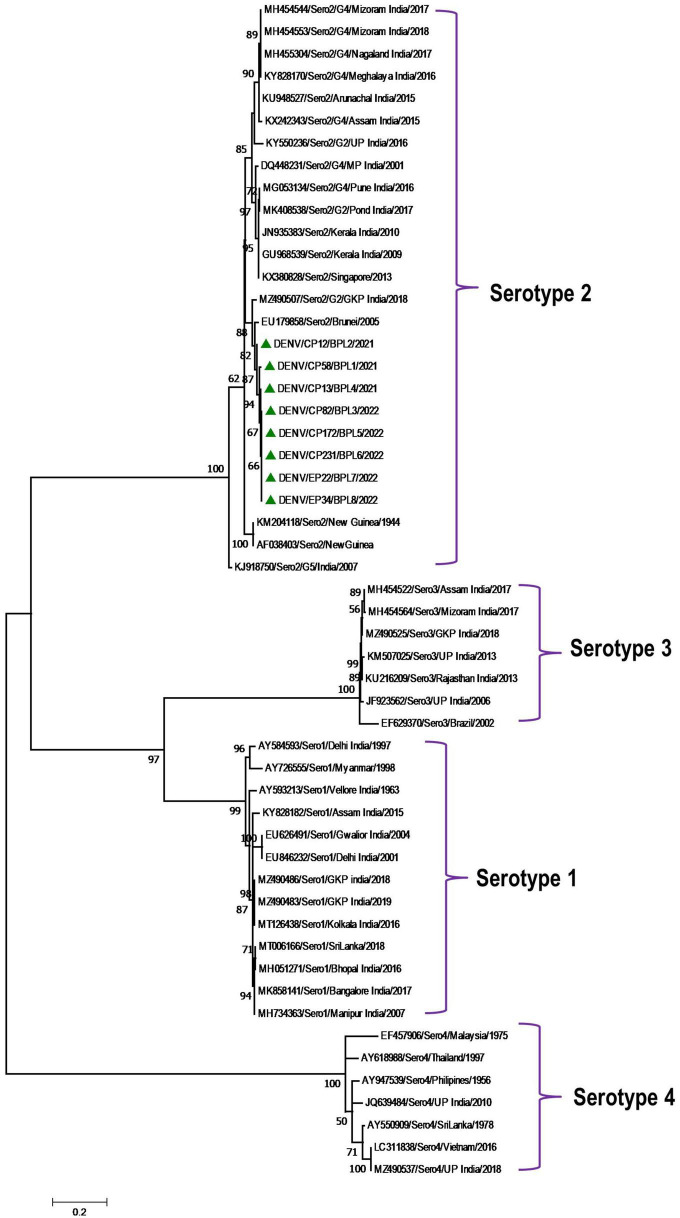
Serotype placement and phylogenetic relationship of DENV isolates using C-prM sequences from the present study and other sequences from India and other countries. The sequences with green colored triangle shape indicates sequence generated in this study. Scale bar indicates number of nucleotide substitutions per site.

**FIGURE 3:**
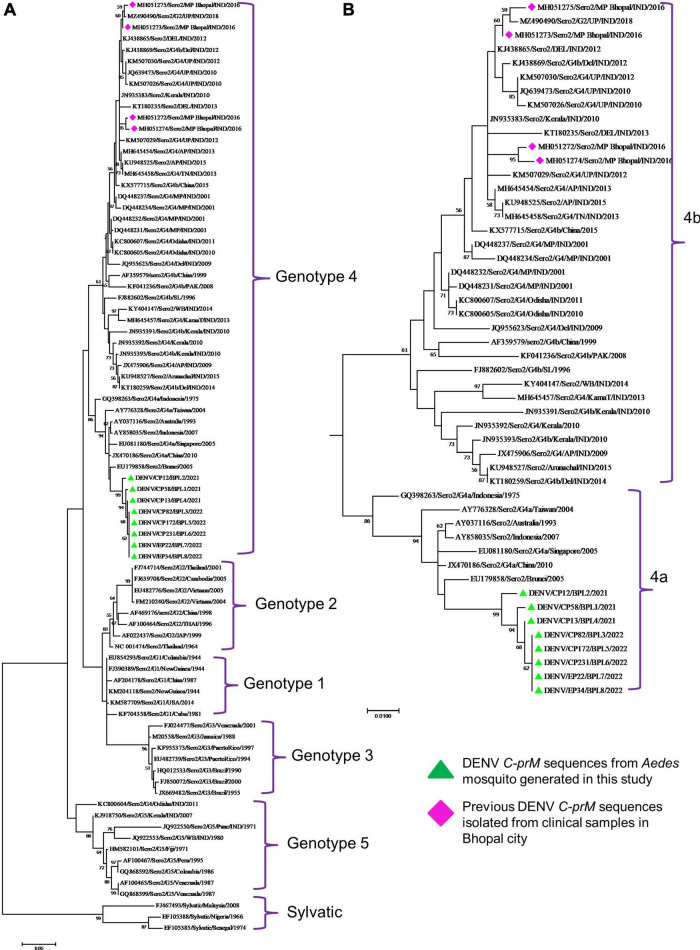
**(A)** Genotypic placement and phylogenetic relationship of DENV-2 isolates using *C-prM* sequences from the present study and from other sequences form India and other countries, **(B)** Phylogenetic tree of DENV-2 corresponding to cosmopolitan genotype (4ab) Sequences. The sequences with green colored triangle shape indicates sequence generated in this study, the sequences with red colored diamond shape indicates previous DENV-2 sequences isolated from clinical samples in Bhopal city. Scale bar indicates number of nucleotide substitutions per site.

**FIGURE 4:**
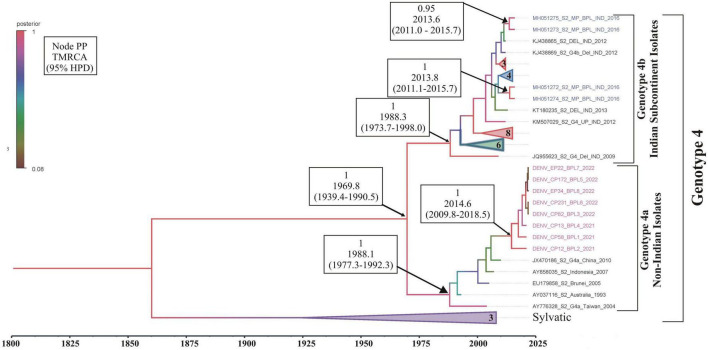
Maximum clade credibility (MCC) phylogenetic tree of DENV-2 corresponding to cosmopolitan genotype (4a,b) generated by Bayesian inference method in BEAST calculated using *C-prM* gene sequences. Sequences in pink represents DENV-2 sequences generated in this study, while sequences in blue are the previous sequences generated from the same study area (Bhopal). All horizontal branch lengths are drawn to a scale of years. PP, posterior probability; TMRCA, the mean estimated time to the most recent common ancestor; 95% HPD, 95% highest posterior density interval.

Phylogenetic analysis revealed that all eight DENV *C-prM* sequences were belonged to serotype 2 (Figure 2). Analysis of the representative serotype 2 *C-prM* sequences from India and other countries unambiguously clustered these 8 sequences with the non-Indian lineage of DENV-2 (Cosmopolitan genotype 4a) (Figures 3A, B). These sequences are substantially divergent from the other genotypes, with nucleotide divergence ranging from 7.4 to 22.2% (Supplementary Figure 2). The maximum clade credibility tree indicated a mean TMRCA of 7.4 years (95% HPD: 3.5–12.2) for the sequences in the present study, suggesting that this lineage was introduced in 2014 (95% HPD: 2009.8–2018.5) (Figure 4). Other isolates collected in Bhopal in 2016 (GenBank accession nos. MH051272-MH051275) grouped into two distinct clades within the most prevalent cosmopolitan 4b genotype, with identical TMRCA (2014, 95% HPD: 2011–2016), implying that both lineages of the DENV-2 cosmopolitan genotype circulated in Bhopal at the same time. When compared to DENV-2 reference strain KM204118 and other DENV-2 sequences, this lineage showed four significant amino acid alterations, namely E19A, M104I, L108M, and D143N (Supplementary Table 2). A statistically significant positive selection was observed in the DENV capsid protein at amino acid position 19 by different methods (MEME: *p*-value 0.05; FEL: *p*-value 0.09; and FUBER: probability 0.962).

## 4. Discussion

Dengue is continuing to be the most serious arboviral infection in various Southeast and South Asian nations, including India. This is due to severe environmental changes, rapid urbanization, and increased human long-distance travel, which have resulted in the spread of *Aedes* species in previously unexplored regions (Hussain and Dhiman, 2022). The first virological dengue outbreak in India was reported in 1963 from Kolkata (East India) (Chatterjee et al., 1965). Both *Ae. aegypti* and *Ae. albopictus* have been identified as potential dengue vectors in different parts of the country, with varied incidences of all four known serotypes (Table 2). Central India, on the other hand, recorded its first dengue outbreak in 1966 in the Jabalpur district of Madhya Pradesh, with DENV-3 as the causative agent. Subsequently, other districts of Madhya Pradesh, such as Sagar (1966), Sarguja (1997), Gwalior (2002), and Bhopal and Indore (2009), also observed dengue outbreaks with DENV 1, 2, and 3 as causative agents (Sehgal et al., 1967; Mahadev et al., 1997; Parida et al., 2002; Kalpana et al., 2010). According to Mahadev et al. (1997), *Ae. aegypti* was the primary cause of the outbreaks in Madhya Pradesh.

**TABLE 2:** Summary of prevalent DENV serotypes and genotypes detected in *Aedes* mosquitoes across the India.

Location	Vector species	DENV serotype and genotype	Minimum infection rate	Period	References
Malkangiri and Angul (Orissa)	*Aedes aegypti* *Aedes albopictus* *Aedes vittatus*	*Aedes aegypti* DENV–2–Genotype IV *Aedes albopictus* DENV–2–Genotype IV DENV–3–Genotype III	NA	2010–2011	Das et al., 2013
Surat (Gujrat)	*Aedes aegypti*	DENV–3–Genotype III DENV–4–Genotype I	NA	2008–2013	Paingankar et al., 2014
Guwahati (Assam)	*Aedes aegypti* *Aedes albopictus*	*Aedes aegypti* DENV–1–Genotype III	*Aedes aegypti*–1.4	2015–2017	Dutta et al., 2018
Guwahati (Assam) Pasighat (Arunachal Pradesh) Tura (Meghalaya) Dimapur (Nagaland)	*Aedes aegypti* *Aedes albopictus*	*Aedes aegypti* DENV–1 DENV–2 DENV–3 *Aedes albopictus* DENV–1 DENV–2	*Aedes aegypti*–11.95 *Aedes albopictus*–35	2018–2019	Chetry et al., 2020
Lucknow (Uttar Pradesh)	*Aedes aegypti* *Aedes albopictus*	*Aedes aegypti* DENV–1–Genotype III and V DENV–2–Genotype IV DENV–3–Genotype III *Aedes albopictus* DENV–1–Genotype III and V DENV–2–Genotype IV DENV–3–Genotype III	*Aedes aegypti*–5.45 *Aedes albopictus*–5.71	2010–2013	Srivastava et al., 2023
Hyderabad (Telangana)	*Aedes aegypti* *Aedes albopictus*	DENV–1–Genotype III DENV–2–Genotype IV DENV–3–Genotype III DENV–4–Genotype I (Species wise is not available)	DENV–1–16 DENV–2–5.33 DENV–3–8 DENV–4–2.66	2017–2018	Sankoju et al., 2023

All four serotypes of DENV have been linked to several outbreaks in India in the past (Guo et al., 2017), however, the majority of these outbreaks were associated with DENV-2 (Singh et al., 1999; Dash et al., 2006; Agarwal et al., 2023). The first DHF outbreak caused by DENV-2 occurred in New Delhi in 1996 (Dar et al., 1999). Since 2012, DENV infections in India have steadily increased, with active circulation of the DENV-2 serotype in different regions with varying prevalence (Sankari et al., 2012; Afreen et al., 2016; Murhekar et al., 2019; Alagarasu et al., 2021). However, the prevalence and distribution of DENV-2 in *Aedes* mosquitoes from Bhopal (Central India) remain unknown. The present study reports for the first time the circulation of DENV-2 serotype and its prevalence in both *Ae. aegypti* and *Ae. albopictus* collected from 29 different localities of Bhopal city. It has been observed that most of the DENV incriminated sites were in the South and South-western part of the city (Figure 1), which were also known to be the hot-spot area of dengue transmission in Bhopal city (Sarma et al., 2022). This finding further confirms the entomological risk for dengue transmission in these areas.

Dengue virus infection in wild-caught *Aedes* mosquitoes was assessed, and circulating DENV serotypes were characterized by sequence comparison and phylogenetic analysis in Bhopal city during the study. This is crucial because molecular characterization of these viruses aids in the identification of molecular subtypes or genotypes and the introduction of any new lineages. Previously, various regions of the dengue genome have been used for molecular phylogenetic analysis, but many studies have reported the *C-prM* gene as a tool in dengue virus genotyping (Murugesan et al., 2020; Titir et al., 2021). We retrieved previously reported *C-prM* gene sequences from the NCBI GenBank database for phylogenetic analysis.

It is evident that cosmopolitan genotypes of the DENV-2 virus are circulating across India (Table 1). However, when analyzing the phylogenetic relationship of DENV sequences generated in this study alongside other global sequences, it became evident that they clustered with non-Indian isolates, indicating the establishment of a novel lineage, lineage 4a, of DENV serotype 2 (Figure 3) in *Aedes* mosquitoes collected from Central India (Bhopal, Madhya Pradesh). Additionally, the MCC tree indicated that this lineage (4a) was introduced in Bhopal around 2013–2014. Previously, the introduction of a new genotype or lineage was proposed as the root cause of the transmission of a severe strain of dengue sickness in other parts of the world, such as the Americas (Rico-Hesse et al., 1997; Messer et al., 2003). It is pertinent to note that Bhopal experienced severe outbreaks of DENV in 2014 and 2016 (Agarwal et al., 2019), and since then the number of dengue cases has been increasing steadily in Bhopal (Sarma et al., 2022). This might be possible because lineages’ extinction and inevitable incursion were connected to a virus’s transmission bottleneck. For example, in Thailand, increased viral transmission by a mosquito vector was also recently connected to lineage replacement (Lambrechts et al., 2012). Similarly, Shrivastava et al. (2015) also reported the new lineage of DENV-3 (genotype 4) in eastern India, which was implicated in the dengue outbreak during the year 2011. However, in view of the scarcity of molecular data on the circulating serotypes/genotypes during that time, pinpointing the lineage that possibly triggered those outbreaks is difficult. The MCC tree shows both the lineages (4a and 4b of the cosmopolitan genotype) were circulating in Bhopal, perhaps with different degrees of dominance. The same has been evidenced by a recent hospital based study in the same area, where out of total 154 RT-PCR positive dengue cases, majority (66 nos.) of them belonged to DENV-2 serotype and of these 66 DENV isolates, 13 were sequenced of which 12 were found to belonged to genotype 4a during 2019 and 2021. It was also observed that the new lineage of DENV-2 was also associated with a longer duration of hospitalization (>10 ± 3 days) and hemorrhagic manifestations indicating its role in severity if disease in the studied area (Agarwal et al., 2023). Higher evolutionary and increased transmission rates of one DENV genotype may outnumber the others in the community, resulting in a lineage shift. This shift may be due to several factors, including changes in the mosquito vector population, host immunity, environmental conditions, and international travel (Shrivastava et al., 2015). Bhopal, a central Indian city, is a favorite destination for international tourists and received 316,195 international travelers during 2014 (India Tourism Statistics, 2014). Although most of the incident dengue cases are of local origin, there are some immigrated cases also, which, along with the international travelers, might facilitates the introduction of this new lineage to the study area.

*Aedes* mosquitoes have the ability to transmit the virus to their offspring, which is identified as vertical transmission. This phenomenon is important to understand as it may contribute to the existence of viruses in the environment in the absence of susceptible hosts. This phenomenon may also be linked to disease endemism, implying that vertical transmission plays an important role in the establishment of endemicity (Hull et al., 1984). The presence and significance of this transmission in *Aedes* have been reported in previous studies from different regions of the world (Martins et al., 2012; Buckner et al., 2013; Wijesinghe et al., 2021). Within India, natural vertical transmission of DENV was observed in dengue hyper-endemic areas such as Rajasthan (Angel and Joshi, 2008; Bina et al., 2008), Tamil Nadu (Arunachalam et al., 2008), New Delhi (Bina et al., 2008), and Kerala (Thenmozhi et al., 2007). Although no DENV infection was observed in the larvae of *Ae. aegypti* and *Ae. albopictus* in the present study, which may be due to low to mid endemicity of the disease in the studied area and low number of larvae processed, the presence of natural vertical transmission of DENV in the studied area couldn’t be ruled out. More molecular surveillance with large numbers larvae is required to confirm the natural vertical transmission of DENV in field collected *Aedes* mosquitoes in this part of India.

## 5. Conclusion

The present study, for the first time, confirms the prevalence of DENV serotype 2 (genotype 4) in field collected *Aedes* mosquitoes from Bhopal city, Central India. The phylogenetic placement of the DENV sequences with non-Indian lineages indicates the circulation of a new lineage (4a) of cosmopolitan genotype. This lineage transition of DENV-2 is a major cause of concern and requires routine surveillance of viral circulation throughout endemic and non-endemic areas for a better understanding of transmission dynamics and effective control and management of the dengue burden in this central part of India.

## Data availability statement

The datasets presented in this study can be found in online repositories. The names of the repository/repositories and accession number(s) can be found in the article/Supplementary material.

## Author contributions

DS: Conceptualization, Formal Analysis, Funding acquisition, Investigation, Methodology, Project administration, Supervision, Writing—original draft. LR: Data curation, Formal Analysis, Investigation, Methodology, Writing—review and editing. SM: Data curation, Formal Analysis, Investigation, Methodology, Writing—review and editing. DD: Investigation, Methodology, Visualization, Writing—review and editing. AA: Formal Analysis, Investigation, Methodology, Visualization, Writing—review and editing. GS: Formal Analysis, Methodology, Writing—original draft. TS: Formal Analysis, Methodology, Writing—review and editing. MKw: Visualization, Writing—review and editing. SaS: Investigation, Writing—review and editing. VV: Visualization, Writing—review and editing. MK: Investigation, Methodology, Writing—review and editing. SwS: Visualization, Writing—review and editing. RT: Supervision, Writing—review and editing. AP: Conceptualization, Methodology, Supervision, Writing—review and editing.
