# Supplementary material for: Molecular surveillance of dengue virus in field-collected Aedes mosquitoes from Bhopal, central India: evidence of circulation of a new lineage of serotype 2
Source: Front Microbiol. 2023 Sep 14;14:1260812. doi: 10.3389/fmicb.2023.1260812 (PMC10539573; doi:10.3389/fmicb.2023.1260812)
Supplement: Supplementary file 1 [file Data_Sheet_1.PDF]

Supplementary material

Figure S1. RT-PCR amplification of *C-PrM* region from field-collected *Aedes* mosquitoes.

Figure S2. Pairwise genetic distance heatmap for all the DENV-2 genotype C-prM sequences. Pairwise mean genetic distances among all the genotypes were shown in the tabulated form within the image.

Table S1. List of DENV serotypes sequences used for construction of phylogenetic tree with GeneBank accession numbers, collection dates, locations, and source of isolation.

Table S2. Amino acid variations between the DENV-2 sequences generated in this study with other DENV-2 cosmopolitan genotype lineage 4a and 4b sequences. Dots represent similar identity of amino acids at different positions on the *C-prM* protein region.

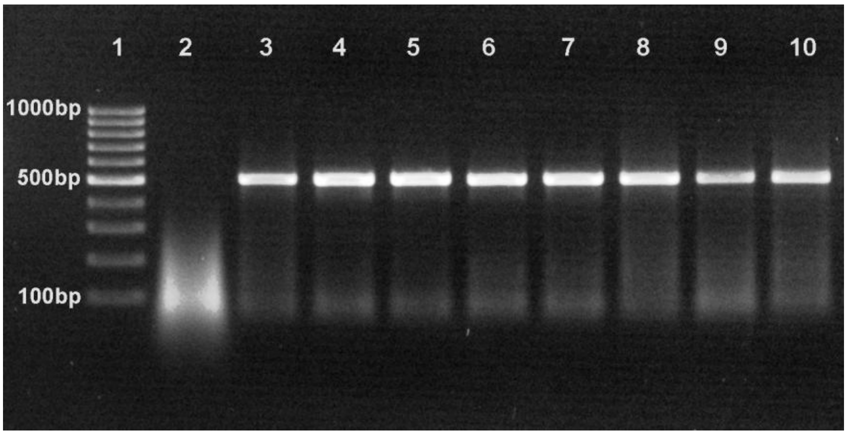

Figure S1. RT-PCR amplification of *C-PrM* region from field-collected *Aedes* mosquitoes. (1: 100 bp DNA marker, 2: negative control, 3-10: DENV positive *Aedes* samples)

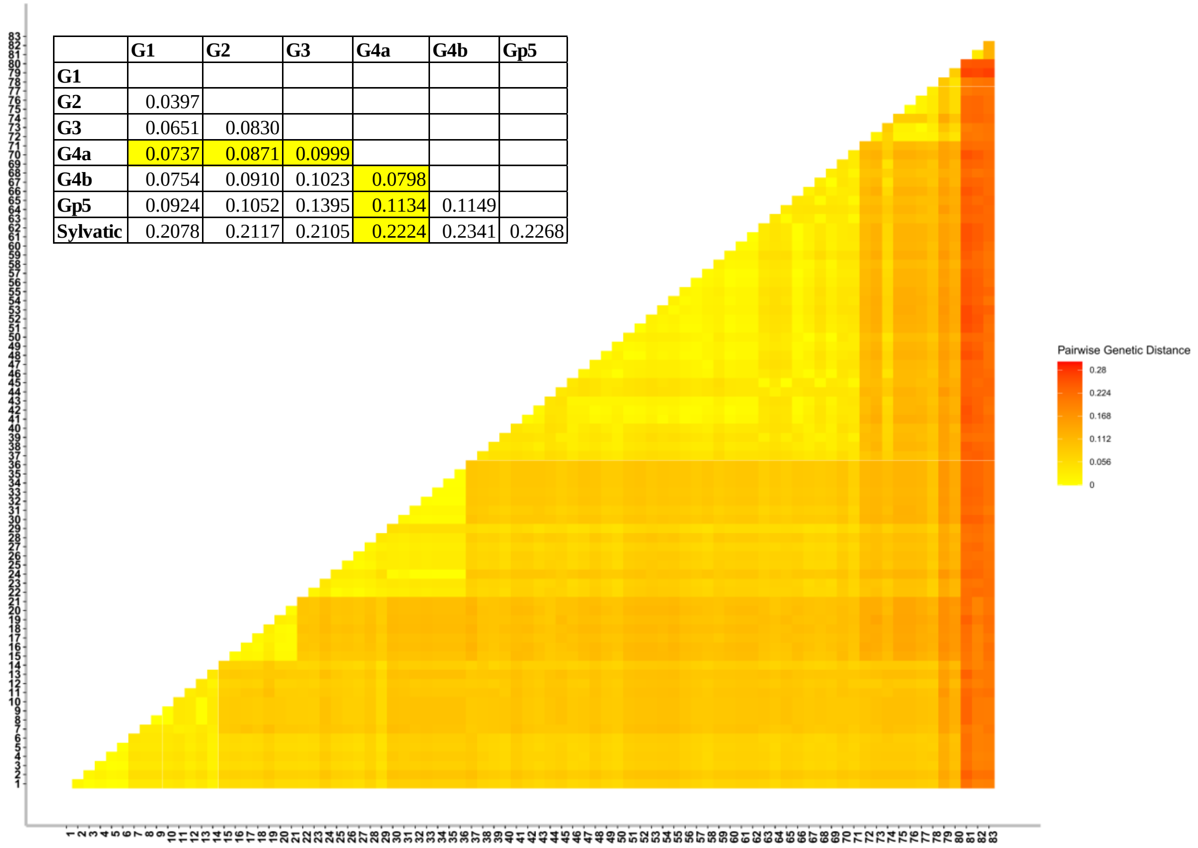

	G1	G2	G3	G4a	G4b	Gp5
**G1**						
**G2**	0.0397					
**G3**	0.0651	0.0830				
**G4a**	0.0737	0.0871	0.0999			
**G4b**	0.0754	0.0910	0.1023	0.0798		
**Gp5**	0.0924	0.1052	0.1395	0.1134	0.1149	
**Sylvatic**	0.2078	0.2117	0.2105	0.2224	0.2341	0.2268

Figure S2. Pairwise genetic distance heatmap for all the DENV-2 genotype C-prM sequences. Pairwise mean genetic distances among all the genotypes were shown in the tabulated form within the image.

Table S1. List of DENV serotypes sequences used for construction of phylogenetic tree with GeneBank accession numbers, collection dates, locations, and source of isolation.

GeneBank Accession no.	Serotype	Location	Year of isolation	Source
AY593213	Sero1	Tamilnadu / India	1963	Homo Sapiens
AY584593	Sero1	Delhi / India	1997	Homo Sapiens
AY726555	Sero1	Myanmar	1998	Not available
EU626491	Sero1	Madhya Pradesh / India	2004	Homo Sapiens
EU846232	Sero1	Delhi / India	2001	Not available
MT006166	Sero1	SriLanka	2018	Homo Sapiens
MH051271	Sero1	Madhya Pradesh / India	2016	Homo Sapiens
MK858141	Sero1	Karnataka / India	2017	Homo Sapiens
MT126438	Sero1	West Bengal / India	2016	Homo Sapiens
MZ490486	Sero1	Uttar Pradesh / india	2018	Homo Sapiens
MZ490483	Sero1	Uttar Pradesh / India	2019	Homo Sapiens
KY828182	Sero1	Assam / India	2015	Homo Sapiens
MH734363	Sero1	Manipur / India	2007	Homo Sapiens
KM204118	Sero2	New Guinea	1944	Homo Sapiens
KJ918750	Sero2	Kerala / India	2007	Homo Sapiens
EU179858	Sero2	Brunei	2005	*Aedes albopictus*
MZ490507	Sero2	Uttar Pradesh / India	2018	Homo Sapiens
KY550236	Sero2	Uttar Pradesh / India	2016	Homo Sapiens
DQ448231	Sero2	Madhya Pradesh / India	2001	Homo Sapiens
MG053134	Sero2	Maharastra / India	2016	Homo Sapiens
MK408538	Sero2	Punducherry / India	2017	Homo Sapiens
JN935383	Sero2	Kerala / India	2010	Homo Sapiens
GU968539	Sero2	Kerala / India	2009	Homo Sapiens
KX380828	Sero2	Singapore	2013	Homo Sapiens
KX242343	Sero2	Assam / India	2015	Homo Sapiens
KY828170	Sero2	Meghalaya / India	2016	Homo Sapiens
MH455304	Sero2	Nagaland / India	2017	Homo Sapiens
MH454544	Sero2	Mizoram / India	2017	Homo Sapiens
MH454553	Sero2	Mizoram / India	2018	Homo Sapiens
AF038403	Sero2	NewGuinea	1998	Not available
KU948527	Sero2	Arunachal Pradesh / India	2015	Homo Sapiens
MZ490525	Sero3	Uttar Pradesh / India	2018	Homo Sapiens
JF923562	Sero3	Uttar Pradesh / India	2006	Homo Sapiens
KM507025	Sero3	Uttar Pradesh / India	2013	*Aedes aegypti*
KU216209	Sero3	Rajasthan_India	2013	Homo Sapiens
EF629370	Sero3	Brazil	2002	Not available
MH454522	Sero3	Assam / India	2017	Homo Sapiens

MH454564	Sero3	Mizoram / India	2017	Homo Sapiens
AY618988	Sero4	Thailand	1997	Not available
EF457906	Sero4	Malaysia	1975	Not available
AY947539	Sero4	Philipines	1956	Homo Sapiens
LC311838	Sero4	Vietnam	2016	Homo Sapiens
MZ490537	Sero4	Uttar Pradesh / India	2018	Homo Sapiens
AY550909	Sero4	SriLanka	1978	Homo Sapiens
JQ639484	Sero4	Uttar Pradesh / India	2010	Homo Sapiens
FJ390389	Sero2	NewGuinea	1944	Homo sapiens
KF704358	Sero2	Cuba	1981	Homo sapiens
EU854293	Sero2	Columbia	1944	Homo sapiens
AF204178	Sero2	China	1987	Homo sapiens
KM587709	Sero2	USA	2014	Not available
AF100464	Sero2	Thailand	1996	Not available
FJ639708	Sero2	Cambodia	2005	Homo sapiens
FJ744714	Sero2	Thailand	2001	Homo sapiens
EU482776	Sero2	Vietnam	2005	Homo sapiens
AF469176	sero2	China	1998	Homo sapiens
AF022437	Sero2	Japan	1999	Not available
FM210240	Sero2	Vietnam	2004	Mosquito
NC_001474	Sero2	Thailand	1964	Not available
FJ850072	Sero2	Brazil	2000	Homo sapiens
JX669482	Sero2	Brazil	1995	Homo sapiens
M20558	Sero2	Jamaica	1988	Not available
KF955373	Sero2	PuertoRico	1997	Homo sapiens
FJ024477	Sero2	Venezuala	2004	Homo sapiens
HQ012533	Sero2	Brazil	1990	Homo sapiens
EU482739	Sero2	PuertoRico	1994	Homo sapiens
AY037116	Sero2	Australia	1993	Homo sapiens
AY776328	Sero2	Taiwan	2004	Not available
AY858035	Sero2	Indonesia	2007	Homo sapiens
JX470186	Sero2	China	2010	Homo sapiens
EU081180	Sero2	Singapore	2005	Not available
GQ398263	Sero2	Indonesia	1975	Homo sapiens
KF041236	Sero2	Pakistan	2008	Homo sapiens
AF359579	sero2	China	1999	Not available
FJ882602	Sero2	Srilanka	1996	Homo sapiens
JQ955623	Sero2	Delhi / India	2009	Homo sapiens
MZ490490	Sero2	Uttar Pradesh / India	2018	Homo sapiens
KJ438869	Sero2	Delhi / India	2012	Homo sapiens
KX577715	Sero2	China	2015	Homo sapiens
KY404147	Sero2	West Bengal / India	2014	Homo sapiens
DQ448234	Sero2	Madhya Pradesh / India	2001	Homo sapiens
JQ639473	Sero2	Uttar Pradesh / India	2010	Homo sapiens
KM507029	Sero2	Uttar Pradesh / India	2012	Aedes aegypti

DQ448232	Sero2	Madhya Pradesh / India	2001	Homo sapiens
KM507030	Sero2	Uttar Pradesh / India	2012	*Aedes albopictus*
KM507026	Sero2	Uttar Pradesh / India	2010	*Aedes albopictus*
MH051274	Sero2	Madhya Pradesh / India	2016	Homo sapiens
MH051275	Sero2	Madhya Pradesh / India	2016	Homo sapiens
MH051272	Sero2	Madhya Pradesh / India	2016	Homo sapiens
MH051273	Sero2	Madhya Pradesh / India	2016	Homo sapiens
DQ448237	Sero2	Madhya Pradesh / India	2001	Homo sapiens
KT180235	Sero2	Delhi / India	2013	Homo sapiens
KJ438865	Sero2	Delhi / IND	2012	Homo sapiens
KU948525	Sero2	Andhra Pradesh / India	2015	Homo sapiens
MH645458	Sero2	Tamilnadu / India	2013	Homo sapiens
JN935393	Sero2	Karela / India	2010	Homo sapiens
MH645457	Sero2	Karnataka / India	2013	Homo sapiens
KT180259	Sero2	Delhi / India	2014	Homo sapiens
MH645454	Sero2	Andhra Pradesh / India	2013	Homo sapiens
KC800607	Sero2	Odisha / India	2011	Mosquito female
JX475906	Sero2	Andhra Pradesh / India	2009	Homo sapiens
KC800605	Sero2	Odisha / India	2010	Mosquito female
JN935391	Sero2	Karela / India	2010	Homo sapiens
JN935392	Sero2	Karela / India	2010	Homo sapiens
HM582101	Sero2	Fiji	1971	Not available
AF100467	Sero2	Peru	1995	Not available
KC800604	Sero2	Odisha / India	2011	Mosquito female
GQ868599	Sero2	Venezuala	1987	Homo sapiens
AF100465	Sero2	Venezuala	1987	Not available
GQ868592	Sero2	Colombia	1986	Homo sapiens
JQ922550	Sero2	Maharashtra / India	1971	Homo sapiens
JQ922553	Sero2	West Bengal / India	1980	Homo sapiens
EF105388	Sero2	Nigeria	1966	Homo sapiens
EF105385	Sero2	Senegal	1974	*Aedes luteocephalus*
FJ467493	Sero2	Malaysia	2008	Not available

Table S2. Amino acid variations between the DENV-2 sequences generated in this study with other DENV-2 cosmopolitan genotype lineage 4a and 4b sequences. Dots represent similar identity of amino acids at different positions on the C-prM protein region.

		**C-Protein**															**prM - Protein**					
	**Amino acid positions**	19	41	63	74	80	92	93	100	102	104	106	108	112	113	143	149	153	163	166	171	174
**Ref Seq**	KM204118	E	R	A	K	V	L	N	R	A	M	I	L	V	M	D	T	M	I	K	R	E
**Lineage 4a**	DENV/CP58/BPL1/2021	A	.	.	R	.	.	.	.	.	I	.	M	.	.	N	.	.	.	N	.	.
	DENV/CP12/BPL2/2021	A	.	.	.	.	.	.	.	.	I	.	M	.	.	N	.	.	.	N	.	.
	DENV/CP82/BPL3/2022	A	.	.	.	.	.	.	.	.	I	.	M	.	.	N	.	.	.	N	.	.
	DENV/CP13/BPL4/2021	A	.	.	.	.	.	.	.	.	I	.	M	.	.	N	.	.	.	N	.	.
	DENV/CP172/BPL5/2022	A	.	.	.	.	.	.	.	.	I	.	M	.	.	N	.	.	.	N	.	.
	DENV/CP231/BPL6/2022	A	.	.	.	.	.	.	.	.	I	.	M	.	.	N	.	.	.	N	.	.
	DENV/EP22/BPL7/2022	A	.	.	.	.	.	.	.	.	I	.	M	.	.	N	.	.	.	N	.	.
	DENV/EP34/BPL8/2022	A	.	.	.	.	.	.	.	.	I	.	M	.	.	N	.	.	.	N	.	.
	JX470186/S2/G4a/China/2010	.	.	.	.	.	.	.	.	.	I	.	M	.	.	N	.	.	.	N	.	.
	AY776328/S2/G4a/Taiwan/2004	.	.	.	.	.	.	.	.	.	V	.	M	.	V	N	.	.	.	N	.	.
	EU179858/S2/Brunei/2005	.	.	.	.	.	.	.	.	.	I	.	V	.	.	N	.	.	.	N	.	.
	AY037116/S2/Australia/1993	.	.	.	.	.	.	.	.	.	I	.	M	.	.	N	.	.	.	N	.	.
	AY858035/S2/Indonesia/2007	.	.	.	.	.	.	.	.	.	I	.	M	.	.	N	.	.	.	N	.	.
**Lineage 4b**	JN935383/S2/Kerala/IND/2010	A	.	.	.	.	.	.	.	.	V	.	.	A	.	.	.	.	.	N	.	.
	KU948525/S2/AP/IND/2015	A	.	.	.	.	.	.	.	.	V	.	.	A	.	.	.	.	.	N	.	.
	DQ448237/S2/G4/MP/IND/2001	.	.	.	.	.	.	.	.	.	V	.	.	A	.	.	.	.	.	N	K	.
	DQ448231/S2/G4/MP/IND/2001	.	.	.	.	I	M	.	.	.	V	.	.	A	.	.	.	.	.	N	.	.
	DQ448234/S2/G4/MP/IND/2001	.	.	.	.	.	.	.	.	.	V	.	.	A	.	.	.	.	.	N	K	G
	KY404147/S2/WB/IND/2014	.	.	.	.	.	.	.	.	.	V	.	.	A	.	.	.	.	.	N	.	.
	KU948527/S2/Arunachal/IND/2015	A	.	.	.	.	.	.	.	V	.	.	.	A	.	.	.	.	V	N	.	.
	DQ448232/S2/G4/MP/IND/2001	.	.	.	.	I	.	.	.	.	V	.	.	A	.	.	.	.	.	N	.	.
	KC800607/S2/G4/Odisha/IND/2011	.	.	.	.	I	.	I	.	.	V	.	.	A	.	.	.	.	.	N	.	.
	KC800605/S2/G4/Odisha/IND/2010	.	.	.	.	I	.	I	.	.	V	.	.	A	.	.	.	.	.	N	.	.
	JN935392/S2/G4/Kerala/2010	A	.	.	.	.	.	.	.	V	.	.	.	A	.	.	.	.	.	N	.	.
	JN935391/S2/G4b/Kerala/IND/2010	A	.	.	.	.	.	.	.	V	.	.	.	A	.	.	.	I	.	N	K	.
	MH645454/S2/G4/AP/IND/2013	A	.	.	.	.	.	.	.	.	V	.	.	A	.	.	.	.	.	N	.	.
	MH645457/S2/G4/KarnaT/IND/2013	A	.	.	.	.	.	.	.	.	.	.	.	A	.	E	.	.	.	N	.	.
	MH645458/S2/G4/TN/IND/2013	A	.	.	.	.	.	.	.	.	V	.	.	A	.	.	.	.	.	N	.	.
	JN935393/S2/G4b/Kerala/IND/2010	A	.	.	.	.	.	.	.	V	.	S	.	A	.	.	.	.	.	N	.	.
	JX475906/S2/G4/AP/IND/2009	.	.	.	.	.	.	.	.	V	.	.	.	A	.	.	.	.	V	N	.	.
	JQ955623/S2/G4/Del/IND/2009	.	.	.	.	.	.	.	.	.	.	.	.	A	.	.	.	.	.	N	.	.
	KJ438869/S2/G4b/Del/IND/2012	-	.	.	.	.	.	.	.	.	V	.	.	A	.	.	.	.	.	N	.	.
